# Cellular and Supracellular Planar Polarity: A Multiscale Cue to Elongate the *Drosophila* Egg Chamber

**DOI:** 10.3389/fcell.2021.645235

**Published:** 2021-03-02

**Authors:** Anna Popkova, Matteo Rauzi, Xiaobo Wang

**Affiliations:** ^1^Université Côte d’Azur, Centre National de la Recherche Scientifique, Institut National de la Santé et de la Recherche Médicale, iBV, Nice, France; ^2^Molecular, Cellular and Developmental Biology Department (MCD), Centre de Biologie Integrative (CBI), University of Toulouse, CNRS, UPS, Toulouse, France

**Keywords:** tissue elongation, planar cell polarity, supracellular network, tissue rotation, actomyosin contractility

## Abstract

Tissue elongation is known to be controlled by oriented cell division, elongation, migration and rearrangement. While these cellular processes have been extensively studied, new emerging supracellular mechanisms driving tissue extension have recently been unveiled. Tissue rotation and actomyosin contractions have been shown to be key processes driving *Drosophila* egg chamber elongation. First, egg chamber rotation facilitates the dorsal-ventral alignment of the extracellular matrix and of the cell basal actin fibers. Both fiber-like structures form supracellular networks constraining the egg growth in a polarized fashion thus working as ‘molecular corsets’. Second, the supracellular actin fiber network, powered by myosin periodic oscillation, contracts anisotropically driving tissue extension along the egg anterior-posterior axis. During both processes, cellular and supracellular planar polarity provide a critical cue to control *Drosophila* egg chamber elongation. Here we review how different planar polarized networks are built, maintained and function at both cellular and supracellular levels in the *Drosophila* ovarian epithelium.

## Introduction

Tissue extension is a fundamental process during embryo development. The extension of tissues contributes to shape the developing embryo and drives the separation of groups of cells that will form different parts of an animal. Unraveling the mechanisms that drive tissue extension is key to understand how life emerges from a cluster of cells. A key and archetypal tissue elongation process is the one directed along the anterior-posterior (AP) axis of the embryo that drives the separation of the head region from the posterior region where the future brain and the anus of the animal will eventually form, respectively (Keller, 2002). Therefore, this primordial shape transformation defines one of the main axes along which the embryo develops and the animal will be structured.

In recent years, a novel mechanism driving *Drosophila* egg chamber elongation has been revealed: supracellular networks, emerging from the coupling of local fiber meshworks and controlled by subcellular planar cell polarity, form around the egg and function as a mechanical corset directing AP egg elongation (Cetera and Horne-Badovinac, 2015). In the first phase of egg elongation, the egg chamber rotates around the AP axis driving extracellular fibers alignment (Haigo and Bilder, 2011; Cetera and Horne-Badovinac, 2015). This results in a polarized extracellular matrix (ECM) working as a passive supracellular scaffold imposing anisotropic boundary conditions during egg chamber growth. In the second phase of egg elongation, a supracellular actomyosin network forms at the basal side of follicle cells (He et al., 2010). Actomyosin fibers run in a direction parallel to the ECM fibrils, and work as an active contractile supracellular scaffold generating polarized mechanical stress to shape the egg chamber. Here we review our current understanding of how supracellular polarized networks form, are maintained and function during *Drosophila* egg chamber elongation.

### Planar Cell Polarity Under the Control of Factor Gradients or Morphogenesis

Epithelial tissues are the fundamental structures forming organs and providing functional shape to multicellular organisms. Epithelial cells show two types of polarity that are orthogonal to one another and that are both required to form and shape tissues: apical-basal polarity (ABP) and planar cell polarity (PCP). ABP is established along the cell apical-basal axis and is necessary to coordinate cell-cell interaction forming the epithelial barriers that separate the inner from the outer side (Harris and Peifer, 2004; St Johnston and Sanson, 2011). PCP is necessary to control the asymmetric organization and reshaping of cells along the plane of an epithelium (Irvine and Wieschaus, 1994; Vichas and Zallen, 2011). PCP pathways often control tissue extension by driving a variety of mechanisms including directed cell division (Baena-Lopez et al., 2005; Aigouy et al., 2010; Campinho et al., 2013), cell elongation (Condic et al., 1991; Nelson et al., 2012; Imuta et al., 2014; Etournay et al., 2015), cell migration (Eaton and Julicher, 2011; Munoz-Soriano et al., 2012) and cell rearrangement (Bertet et al., 2004; Blankenship et al., 2006). For tissues presenting an either open or closed topology (e.g., the wing disk or the egg chamber, respectively), PCP results in polarized signals under the control of factor gradients. Well-known examples are the Frizzled/Strabismus and Fat/Dachsous PCP signaling pathways that, for instance, play key roles in the development of the *Drosophila* eye and wing (Brittle et al., 2010, 2012; Goodrich and Strutt, 2011). In addition to the controlling factor gradients, global PCP patterns can also be under the control of cell and tissue mechanics governed by actomyosin contractility and cell-cell adhesion driving tissue movement and flow during morphogenesis. A remarkable example is the one shown during the *Drosophila* fly wing development where tissue movement and morphogenesis guide PCP (Aigouy et al., 2010). This demonstrates that PCP can be not only the cause but also the consequence of epithelial morphogenesis. In the *Drosophila* egg chamber, the PCP core system relies upon the Fat/Dachsous signal transduction pathway (Gutzeit et al., 1991). In this topologically closed epithelium, the global PCP pattern is also under the control of tissue movement: the egg chamber rotation around the AP axis guide PCP, building polarized ECM (Haigo and Bilder, 2011).

### *Drosophila* Egg Chamber Elongation Is Driven by Molecular Corsets

**FIGURE 1:**
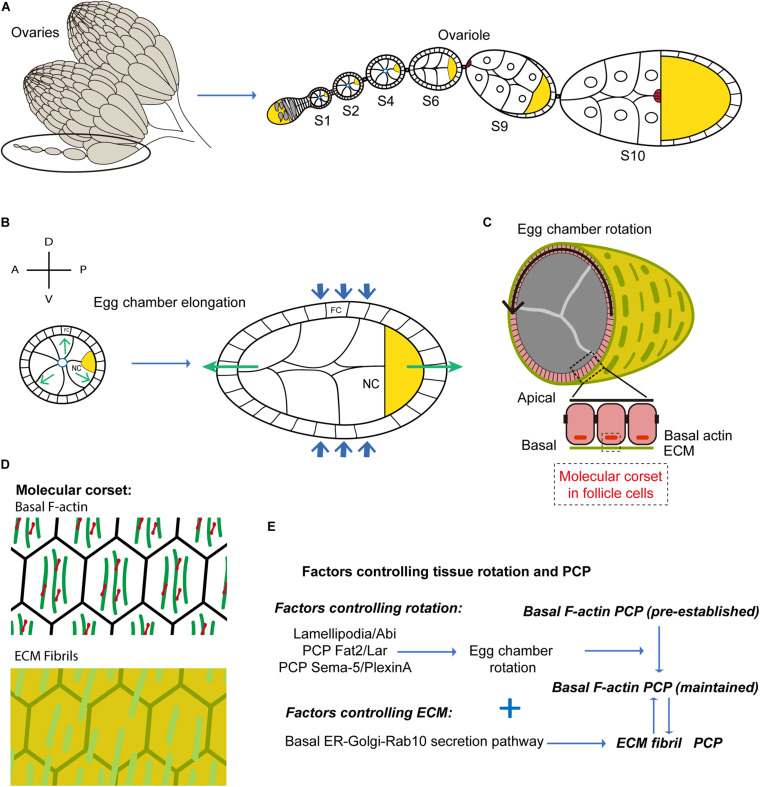
Egg chamber rotation and planar cell polarity during *Drosophila* oogenesis. **(A)** Schematic diagram illustrating *Drosophila* ovary, composed of 15–20 ovarioles. Each ovariole is composed of a string of egg chambers at different developmental stages. **(B)** Schematic diagram illustrating egg chamber morphology at stages 5–8. FC, follicle cells; NC, nurse cells; oocyte in yellow. Anterior-posterior (AP) and Dorsal-ventral (DV) axes are indicated. Green arrows mark the direction of tissue expansion, and blue arrows mark the corset-restrictive forces. The egg chambers expand their volume from early to late stages. With the corset-restrictive forces, the egg elongates along the AP-axis during their expansion processes. **(C)** Schematic representation of the cross-sections of egg chamber at stages 5–8. Arrows indicate the sense of tissue rotation. **(D)** Schematic representation of the molecular corsets at stage 5–8. The molecular corsets are composed of parallel fibrillary ECM at the BM and parallel actin bundles at the basal domain of follicular epithelial cells. **(E)** Schematic diagram showing the mechanisms driving tissue rotation and planar cell polarity during *Drosophila* oogenesis.

During *Drosophila* oogenesis, the egg undergoes maturation before being expelled from the ovary and laid by the mother. The *Drosophila* ovary is composed of 15–20 ovarioles (Figure 1A). Each ovariole contains a linear sequence of egg chambers at 14 different developmental stages connected to one another via linking cells forming stalks (Figure 1A; He et al., 2011). Germline and somatic stem cells reside near the tip of the ovariole, a region named the germarium. These two stem cell types assemble forming an egg chamber budding off from the germarium. The perpetual serial assembly of egg chambers results in the formation of a linear alignment of interconnected chambers at different developmental stages, resembling ‘pearls on a string’. The egg chamber is composed of a monolayer follicular epithelium surrounding a 16-cell germline cyst (He et al., 2011). The apical side of the follicular epithelium makes contact with the internal germ cells, while the basal side interacts with the extracellular basement membrane (BM). During oogenesis, the egg chamber gradually changes its shape from spherical to ellipsoidal by extending along the AP axis (Figure 1B). The egg chamber elongation rate is moderate between stage 1 (S1) and S4, while it is higher between S5 and S11 (Haigo and Bilder, 2011) eventually resulting in an oblong shaped embryo at full maturation.

Egg chamber elongation is driven by polarized ‘molecular corsets’ that wrap and constrain the egg along its shorter axes directing egg chamber growth (Figure 1C). These corsets are composed of parallel fibrillary ECM at the BM and parallel actin bundles forming stress-fibers at the basal domain of follicular epithelial cells (Figure 1D). Both ECM and actin bundles form polarized networks at the surface of the egg - an enveloping scaffold orthogonal to the AP axis (Figure 1D; Cetera and Horne-Badovinac, 2015). During S5 to S8, the ECM works as a passive scaffold resisting the expansive growth of internal germ cells. Egg chamber volume increase is thus biased toward the poles resulting in AP egg extension. Crest et al. (2017) used atomic force microscopy to measure BM stiffness of the egg chamber at different stages during egg chamber rotation and at different AP positions. Interestingly, they showed that BM stiffness increases during egg chamber maturation and that a stiffness gradient is established along the AP axis with highest stiffness in the central zone. BM differential stiffness is under the control of JAK/STAT signaling and is necessary to impose differential resistance to isotropic tissue expansion resulting in egg chamber elongation (Crest et al., 2017).

During S9 and S10, the actin bundles contract under the action of the myosin (MyoII) motor protein to form an active contracting scaffold that directs egg extension (Popkova et al., 2020). How are polarized scaffolds formed? More precisely, how do actin bundles and ECM fibrils align? Actin bundle alignment is pre-established at very early stages of oogenesis (region 2b) (Chen et al., 2016) and the controlling factors are not known. In contrast, ECM fibril formation takes place between S2 and S8 via an atypical mechanism based on egg chamber polarized rotation around the AP axis.

### Tissue Rotation and Planar Cell Polarity During *Drosophila* Oogenesis

Between S2 and S8, the egg chamber rotates along the AP axis (Figure 1C). Since six to seven egg chambers align to form a ‘string’, four to five chambers rotate simultaneously within each ovariole. Interestingly, while the rotation velocity is overall higher at later stages (with the exception of S8 when the egg rotation slows down), the angular velocity is rather constant between S3 and S7 (Chen et al., 2016) despite the >10-fold increase in egg volume (Chen et al., 2016, 2019). Egg chamber rotation is driven by the directed collective movement of follicular epithelial cells (Figure 1E). Since the follicle cells adhere to the internal germ cells, follicle cell collective migration results in the rotation of the entire egg chamber (Haigo and Bilder, 2011; Cetera and Horne-Badovinac, 2015). Remarkably, ECM fibrils lengthen and align along the direction of follicle cell rotation (i.e., egg chamber rotation). How do ECM fibrils form along planes orthogonal to the AP axis to form a supracellular molecular corset? Follicle cells, during rotatory migration, secrete BM components forming ECM fibril stripes at the rear, much like a ‘snail slime trail’ (Chen et al., 2017). BM components are synthesized in a basally located endoplasmic reticulum (ER) compartment and are then transported to the basal Golgi (Lerner et al., 2013). A Rab10-dependent secretory pathway controls the secretion of newly synthesized proteins in the pericellular space between follicular epithelial cells (Isabella and Horne-Badovinac, 2016). As a consequence of the directed movement of follicle cells, the secreted proteins are then directionally inserted into the BM (Isabella and Horne-Badovinac, 2016). The coordination between the Rab10-based secretion pathway and the directed collective cell movement, guarantees the formation of polarized BM fibrils enveloping the follicular epithelium (Figure 1E). Finally, in the Fat2 loss-of-function mutants in which tissue rotation (i.e., directed cell migration) is hampered, ECM fibrils form disorganized patterns (Lerner et al., 2013).

What are the cellular mechanisms and the signaling factors controlling the directed collective movement of follicle cells? Actin-based protrusions, formed at the follicle leading edge, often can function as exploratory antennas or traction force generators directing cell movement (Gardel et al., 2010; Ridley, 2011). Both filopodia and lamellipodia (i.e., typical exploratory and force generation cell protrusive structures, respectively), have been observed in the leading edges of migrating follicular epithelial cells during early stages of egg chamber rotation (Lewellyn et al., 2013; Cetera et al., 2014). Lamellipodia formation and tissue rotation are under the control of the SCAR/Wave complex, an activator of the actin nucleator Arp2/3 that is necessary to establish the dynamic branched actin network propelling the lamellipodium (Figure 1E; Cetera et al., 2014). Nevertheless, it is still not clear whether other critical factors controlling lamellipodia formation [e.g., the small GTPase Rac1 (Ridley et al., 1992; Wang et al., 2010)] play a role in this process. Recent studies have identified two PCP signaling pathways coordinating the leading and the trailing edge dynamics between neighboring follicle cells (Figure 1E; Barlan et al., 2017; Stedden et al., 2019). One PCP system is mediated by the atypical cadherin Fat2 and the Leukocyte-antigen-related-like (LAR) receptor tyrosine phosphatase (Barlan et al., 2017). These two proteins participate in the formation of protrusions in a non-cell-autonomous manner. The Fat2 based signals of follicular epithelial cells are located at the cell trailing edge inducing the formation of leading-edge protrusions in the cells located at the ‘rear’. This protrusion induction mechanism is partially mediated by Lar stabilization at the leading edge of the rear cell. This results in the coordinated retraction and extension of the trailing edge of the cell at the front and of the leading edge of the cell at the rear, respectively. A second PCP system has been uncovered recently: this is based on the Semaphorin-5c and PlexinA factors providing additional coordination control over leading and trailing edges (Stedden et al., 2019). The SCAR/Wave and the PCP (Fat2/Lar and Semaphorin-5c/PlexinA) pathways, both coordinating the interface between the front and rear cells, could potentially synergize to orchestrate the directed collective cell migration (Figure 1E).

Actin bundles at the basal side of follicle cells align orthogonally to the AP axis from very early stages before egg chamber formation and during germarium development. This pre-polarized network could function as a cue to initiate directed follicle cell migration. While egg chamber rotation is thus the original cause of ECM fibril but not actin bundle polarity, rotation was shown to be responsible for the maintenance of actin bundle polarity during egg chamber maturation (Chen et al., 2016).

Egg chamber rotation stops at early stage 9, when the oscillatory contraction of the follicle basal actomyosin network starts to guide the second phase of egg chamber extension.

### Oscillatory Actomyosin Contractions and Planar Cell Polarity During *Drosophila* Oogenesis

**FIGURE 2:**
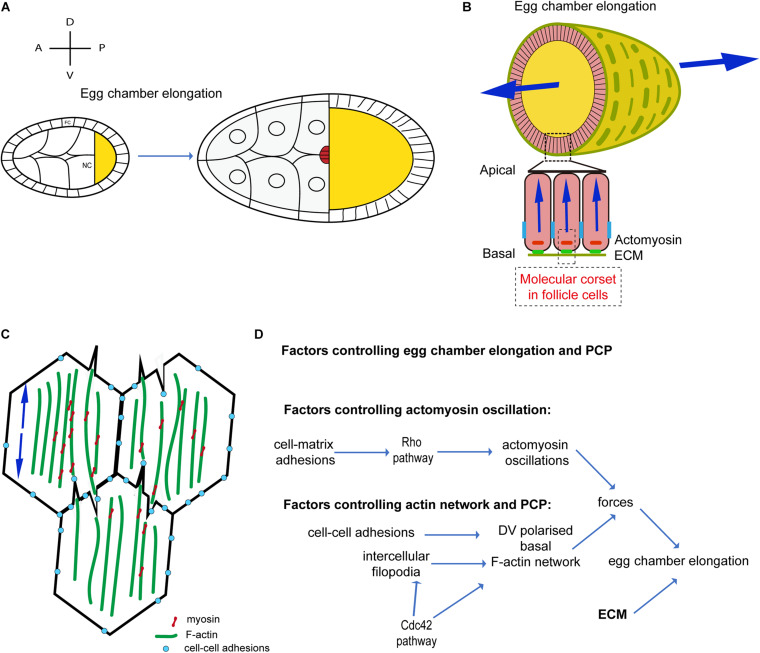
Oscillatory actomyosin contractions and planar cell polarity during *Drosophila* oogenesis. **(A)** Schematic diagram illustrating egg chamber morphology at stages 9–10. AP and DV axes are indicated. FC, follicle cells; NC, nurse cells; oocyte in yellow. The egg chamber elongates along the AP-axis. **(B)** Schematic representation of the cross-sections of the egg chamber at S9–S10. Arrows indicate forces directed along the AP axis. **(C)** Schematic representation of the actomyosin distribution at the basal side of follicle cells in contact during S9–S10, showing filopodia penetrating the actomyosin cortex of a neighboring cell and forming a supracellular contractile network. Arrows indicate contractile forces. **(D)** Schematic diagram showing the mechanisms driving actomyosin contractions and planar cell polarity during *Drosophila* oogenesis.

During S9 and S10 (Figure 2A), after egg chamber rotation arrest, MyoII medial-basal oscillations contract follicle cell stress-fibers driving periodic constriction of follicle cell basal surface and further extending the egg chamber (He et al., 2010; Figure 2B). Basal MyoII oscillations depend on the Rho1-ROCK pathway and on cell adhesion. Rho1 is strongly enriched at the basal junctions of follicle cells during S9 and S10 and it is positively regulated by cell-matrix but not cell-cell adhesion (Qin et al., 2017). Under the control of Rho1, ROCK is upregulated at the basal junctional cortex and eventually concentrates at the cell medial-basal region (Qin et al., 2018). Basal actomyosin periodic contractions reduce the follicle cell basal area along the dorsal-ventral (DV) direction (He et al., 2010), supporting the hypothesis that basal actomyosin networks drive DV polarized tension, remodeling basal surface area. In a recent study, stress-fiber tension anisotropy, at the basal side of follicle cells, was directly probed and measured by implementing infrared (IR) femtosecond (fs) laser ablation to dissect the actomyosin cytoskeleton with subcellular precision (Popkova et al., 2020). During S9, tension in the medial-basal network of follicle cells is an order of magnitude higher along the DV axis compared to the AP axis. Basal area fluctuations do not result in a rapid ratcheted net surface reduction since (i) both levels of fiber actin (F-actin) and MyoII oscillate without net increase and (ii) contractions are non-synchronous between cells (He et al., 2010). This process shows similarities with the periodic surface oscillations reported in dorsally located cells during amnioserosa contractions (Solon et al., 2009) or in ectoderm cells during germband extension (Vanderleest et al., 2018). Follicle cell basal contraction is instead in net contrast with cell apical constriction in ventral furrow formation during *Drosophila* embryo gastrulation where cell-cell concerted net accumulation of MyoII ensures rapid and progressive cell apical surface reduction (Martin et al., 2009, 2010). Rho1 pathway upregulation in the follicular tissue results in MyoII oscillation arrest and basal MyoII net accumulation driving acute tissue AP extension (He et al., 2010; Qin et al., 2017). This evidence supports the idea that actomyosin periodic oscillations, devoid of MyoII net accumulation, ensure gradual tissue shape change over longer periods of time and avoid abrupt morphological transition. During S9, stress-fiber anisotropic tension could oppose polarized resistance to the gradual volume increase of the egg chamber directing tissue expansion toward the poles (similarly to the ECM at earlier stages).

Tissue morphogenesis relies upon cell-cell mechanical coupling for force transmission (Guillot and Lecuit, 2013; Heisenberg and Bellaiche, 2013). Mechanical coupling is usually mediated by adherens junctions: cell-cell adhesive sites are constituted by a dense F-actin network coupled to E-cadherin proteins (Ratheesh and Yap, 2012; Lecuit and Yap, 2015). Remarkably, between S9 and S10, parallel F-actin fibers span across the medial-basal side of follicle cells and are not specifically enriched at junctions where low levels of mechanical tension are reported (Santa-Cruz Mateos et al., 2020). In addition, MyoII is absent at cell-cell junctions and it accumulates solely in the cortical medial-basal region. How are forces transmitted between follicle cells to establish tissue scale contractile tension? Supracellular F-actin bundles form across cells mediated by bi-directionally polarized filopodia (Popkova et al., 2020; Figure 2C). These long cell extensions, eventually culminating with E-cadherin based junctions, reciprocally interdigitate penetrating the actomyosin cortices of opposing cells. Follicle cell filopodia are tension sensitive: IR fs dissection of stress-fibers juxtaposed to filopodia induce tension release and filopodia retraction. In addition, filopodia can mediate cell-cell coupling eventually functioning as mechanical anchors bridging cell cortexes (Popkova et al., 2020). Santa-Cruz Mateos et al. (2020) report that F-actin protrusions increase in follicle cells neighboring integrin-mutant cells. Further work is necessary to better elucidate how filopodia protrusions interact with stress-fibers of neighboring cells and how this unusual cell-cell interaction mechanisms can cross-talk with cell-matrix adhesion.

Supracellular IR fs laser ablation of the actomyosin cortex, along a line parallel to the AP axis, results in one single large opening rather than multiple cell-to-cell independent F-actin network recoils. This demonstrates that supracellular tension results from the coupling of local cellular contractions. In addition, actomyosin dissection along a line parallel to the DV axis results in much slower recoil compared to a linear dissection along the orthogonal direction (i.e., the AP axis). This shows that the egg chamber during S9-S10 is under anisotropic tension with higher tension along the DV axis (Popkova et al., 2020). Both intercellular filopodia and stress-fibers are under the control of the activity of the small GTPase Cdc42. Genetic as well as optogenetic inhibitions of Cdc42 activity lead to reduced filopodia length and misaligned stress-fibers resulting in lower cell medio-basal tension (Popkova et al., 2020). Cdc42 is thus a key player ensuring tension anisotropy at the cellular scale. Furthermore, Cdc42 also plays a non-cell autonomous role in stress-fiber alignment and cell tension anisotropy. In follicular epithelia containing large Cdc42DN clones, tissue scale tension is drastically reduced along the DV axis: in Cdc42 mutants, the follicular epithelium fails to extend, thereby resulting in a more round-shaped egg chamber. This shows that the supracellular stress-fiber network, under the control of Cdc42, is powered by basal MyoII oscillations and drives anisotropic tension for tissue extension.

How is stress-fiber polarity established and maintained? F-actin stress-fibers align along the DV axis at very early stages of oogenesis (region 2b) under the control of an unknown factor. Stress-fiber alignment is eventually maintained during S9-S10 under the control of cell-cell adhesion and Cdc42 (Qin et al., 2017; Popkova et al., 2020). When E-cadherin cell-cell adhesion is downregulated, actin stress-fibers relocate to the basal junctional cortex and both ROCK and MyoII expand their localization more apically (Qin et al., 2017). When Cdc42 is inhibited, stress-fibers misalign and lose their DV polarity. Intercellular filopodia, under the control of Cdc42, could play a key role in maintaining stress-fiber alignment after egg chamber rotation arrest (S9 and S10) (Figure 2D).

Cell-ECM adhesions could also play a role in the organization of stress-fibers at the basal side of follicle cells since the inhibition of cell-matrix adhesion by RNAi, optogenetics, or loss-off-function alleles can eventually affect F-actin and MyoII levels and/or distribution in the basal region of follicle cells (Qin et al., 2017; Santa-Cruz Mateos et al., 2020). Further work is necessary to better elucidate the role of adhesion between the follicle cells and the ECM to establish a polarized F-actin supracellular network. Santa-Cruz Mateos et al. (2020) reported increased membrane tension in an integrin-mutant background. While more appropriate tools/protocols need to be implemented to discern between membrane and cytoskeletal tension at the cortex, it would be interesting in the future to probe stress-fiber tension in an integrin-mutant background to better understand the role of integrin-based adhesion to drive tension anisotropy at both cellular and supracellular levels. In a recent study, Cerqueira Campos et al. (2020) explored the role of the Dystroglican-associated protein complex (DAPC) in controlling ECM and F-actin network formation and polarity. DAPC is a transmembrane complex that links ECM to the F-actin cytoskeleton during follicle tissue elongation. The two main components of the DAPC [Dystrophin (Dys) and Dystroglycan (Dg)] are required during early oogenesis for follicle elongation and proper BM fibril and stress-fiber formation, but neither for rotation nor for the initial establishment of Fat2 PCP. Finally, the authors proposed that DAPC-dependent BM deposition at earlier stages (until S7) functions as a PCP ‘memory’ for F-actin stress-fiber alignment at a later stage (S13).

The spatial pattern of MyoII oscillations can be divided into three phases: (1) an initiation phase during early S9 (ES9) when MyoII oscillations appear in the anterior 1/3 region of the egg chamber; (2) a propagation phase between middle S9 (MS9) and beginning of S10 (S10A) when MyoII oscillations spread by shifting posteriorly and increasing in amplitude; and (3) a stabilization phase during middle to late of S10 (S10B) when all follicle cells contacting the oocyte accumulate medial-basal MyoII and MyoII oscillations reduce in amplitude maintaining high intensity levels. How the MyoII spatiotemporal pattern is controlled is still poorly understood. Koride et al. (2014) proposed a mechano-transduction-based mechanism that controls MyoII spatio-temporal patterns. This model was then challenged in 2018 by Qin et al. (2018), who showed that MyoII oscillation are tension independent.

## Discussion

Life often starts from a shapeless cluster of cells. Tissue elongation is among the very first morphogenetic processes to mold this cellular cluster during embryo development. Studying the fundamental mechanisms driving epithelial extension is thus a very exciting opportunity that will help us understand the emergence of life. In this review, we focus on the process of elongation in the *Drosophila* egg chamber, a powerful model system to study the cellular mechanisms driving epithelial morphogenesis.

Over the last decade, a new mechanism driving tissue elongation has been unraveled: polarized forces, driven by a molecular corset, work to extend the egg chamber. The molecular corset is a two-tier wafer-like scaffold: F-actin stress-fibers at the basal side of follicle cells underlay polarized ECM fibrils at the BM. Both fiber networks are DV polarized and are wrapped around the egg chamber. While during the first phase of elongation (until S8) the corset works as a passive scaffold, during the second phase (S9-S10) the corset generates active forces powered by actomyosin contractions. F-actin stress-fibers are pre-polarized in the germarium, while ECM fibril polarization is under the control of egg chamber rotation around the AP axis during S2 to S8. Egg chamber rotation is driven by follicle cell collective migration: follicle cells undergo rounds of revolution propelled by polarized lamellipodia. The ECM secreted by follicle cells during migration results in the formation of DV aligned fibrils. Which mechanism controls the direction of follicle cell collective migration? Egg chambers are connected to each other via stalk cells at the anterior and posterior poles forming a pearl-string-like structure. These polar connecting sites could bias the direction of follicle cell migration by limiting the egg-chamber free degrees of rotation. If the direction of migration is imposed by the boundary conditions of the system, it is still not clear which mechanism controls the sense of egg chamber rotation. Egg chambers belonging to the same ‘string’ can rotate both in a clockwise and anti-clockwise direction (Haigo and Bilder, 2011). Under the control of stochastic subcellular polarity, one or a group of cells could take the lead by imposing a directional sense of movement (Jain et al., 2020). A mechanism based on stochastic polarity could be investigated by using optogenetics to induce or inhibit cell protrusion formation in a spatiotemporal specific fashion.

Egg chambers rotate with equal angular velocity during S3 to S7. Constant angular velocity at different egg-chamber stages can be achieved if follicle cells finely tune their speed of migration in a way that is directly proportional to the egg-chamber radius. Strong mechanical coupling between egg chambers belonging to the same ‘string’ could explain how angular velocity is conserved between egg chambers of different sizes: the rotation of one egg chamber would impose the same angular velocity to the other neighboring egg chambers (like wheels of different sizes mounted on the same axle). Nevertheless, this hypothesis may be ruled out since egg chambers on the same string may rotate in opposite directions. Future work is necessary to unveil the mechanisms controlling follicle cell migration imposing constant angular velocity to egg chambers at different stages.

The egg chamber is an ovoid structure. If all cells of one egg chamber migrate around the AP axis with the same angular velocity, cells located at the poles must migrate at slower speed than cells at the equator. How is cell speed finely controlled at different egg-chamber AP positions? Follicle cell collective migration could result from a cell leader-follower based mechanism (Barlan et al., 2017; Jain et al., 2020): for instance, medial follicle cells, that are more numerous, may act as leaders generating greater driving force. The central regions, under the control of JACK/STAT, is the portion of the egg chamber with denser ECM (Crest et al., 2017). Follicle cells located medially could thus adhere more strongly to the ECM generating greater traction forces to power migration. The denser ECM in the medial portion of the egg chamber could also be, at the same time, the result of a greater number of follicle cells located medially and secreting ECM during migration.

Stalk cells form connecting regions between egg chambers. If neighboring egg chambers rotate in the opposite sense, stalks cells would experience torsion opposing resistance to rotation. Cell intercalation between stalk cells and follicle cells at the egg-chamber poles is a mechanism that could facilitate counter-rotation between neighboring egg chambers. Future work is necessary to better elucidate the role of stalks during egg chamber rotation.

The periodic contraction of the cell actomyosin cytoskeleton is a process usually reported during cell shape changes and tissue morphogenesis (Munro et al., 2004; Skoglund et al., 2008; Martin et al., 2009; Solon et al., 2009; He et al., 2010; Rauzi et al., 2010). The biochemical oscillators controlling basal or apical MyoII recruitment share common features: periodic accumulation of Rho1 and ROCK control the dynamics of Myo-II recruitment by periodically phosphorylating the MyoII regulatory light chain. With similar dynamics, phosphatases periodically trigger MyoII dephosphorylation (Tan et al., 2003; Kasza et al., 2014; Simoes Sde et al., 2014; Vasquez et al., 2014; Munjal et al., 2015; Mason et al., 2016; Banerjee et al., 2017; Belmonte et al., 2017; Chanet et al., 2017; Michaux et al., 2018; Senger et al., 2019). Apical and basal periodic actomyosin contraction also show striking differences. At the cell apical-medial side, F-actin forms a cortical meshwork coupled to adherens junctions that can flow under MyoII load generating cortical advection (Rauzi et al., 2010; Munjal et al., 2015). The actomyosin flow induces a transient increase of actomyosin density that is referred to as ‘pulse’. At the basal side of follicle cells, F-actin forms a network of parallel stress-fibers linked to the ECM via integrin-mediated adhesion (He et al., 2010; Qin et al., 2017). The F-actin network under MyoII load is able to contract along the direction of the stress-fibers but not to flow (Qin et al., 2017). The periodic contraction of actomyosin stress-fibers is referred to as ‘oscillations’. ‘Pulses’ and ‘oscillations’ have the common feature of being periodic. Periodic constrictions could facilitate the gradual change in cell shape avoiding cell jamming or aberrant acute tissue shapes. MyoII oscillations in follicle cells appear asynchronous (He et al., 2010). It is still not clear whether oscillations of neighboring cells are coupled one another. In embryo wound repair it has been suggested that actomyosin contractility in discrete segments of the wound edge signals through a stretch-activated ion channel (SAIC) to neighboring segments to promote actomyosin assembly and to coordinate wound repair (Zulueta-Coarasa and Fernandez-Gonzalez, 2018). MyoII was also reported to accumulate under an ectopic mechanical stimulus (Fernandez-Gonzalez et al., 2009; Pouille et al., 2009) or endogenous cell stretching (Bailles et al., 2019). A mechano-transduction pathway could also be activated between neighboring follicle cells during actomyosin oscillation. The follicle cells located anteriorly and enveloping the nurse cells do not exhibit basal MyoII oscillations. During S9, follicle cells change shape from cuboidal to either squamous (enveloping the nurse cells) or columnar (enveloping the oocyte) (Kolahi et al., 2009). Cell geometry might thus play a role in actin cytoskeleton organization and actomyosin oscillations (Vignaud et al., 2012). The cross-talk between F-actin stress-fibers and ECM is still unclear. During S2-S8, the stress-fiber network could work in tandem with the ECM fibrils to form a passive scaffold resisting the egg chamber expansion. During S9-S10, the ECM could contribute to integrate, at the tissue scale, periodic cellular constricting forces generated by the actomyosin network. These are key hypotheses that need to be tackled in the future.

## Author Contributions

XW and AP prepared the figures. MR commented on the figure revision. All authors wrote and revised the manuscript.

## Conflict of Interest

The authors declare that the research was conducted in the absence of any commercial or financial relationships that could be construed as a potential conflict of interest.
